# Efficacy of Modified Masood Scoring System (MMSS) in Cytological Diagnosis of Breast Lesions

**DOI:** 10.7759/cureus.22296

**Published:** 2022-02-16

**Authors:** Justina William, Kanwal Masih, Uma Pradhan, Jasneet Kaur, Upinder Singh, Gulshanjit Singh

**Affiliations:** 1 Department of Pathology, Christian Medical College and Hospital, Ludhiana, IND; 2 Department of General Surgery, Shree Guru Gobind Singh Tricentenary (SGT) University, Gurugram, IND; 3 Department of Pathology, Genomics Laboratory, New Delhi, IND

**Keywords:** proliferative/nonproliferative breast lesions, diagnosis difficulty, breast histopathology, modified masood scoring system, fnac breast

## Abstract

Introduction: Fine-needle aspiration cytology (FNAC) breast is generally considered a reliable diagnostic tool to distinguish non-proliferative from proliferative breast lesions. Nevertheless, differentiating these breast lesions on FNAC poses a diagnostic challenge. Modified Masood Scoring System (MMSS) based on cytomorphological examination has been used to help in differentiating these lesions.

Material and methods: A total of 67 patients were included in this prospective study done from November 2012 to May 2014 and the breast lesions were cytologically categorized by conventional and as per MMSS criteria, followed by comparison to a histopathological examination, which was taken as a gold standard. Relevant frequencies and proportions were calculated along with the sensitivity and specificity of the MMSS.

Results: The age of the patients ranged from 15 to 85 years with a mean age of 44.3 ± 14.8 years. Females predominated in the study and right-sided breast lesions were more common compared to the left side. Overall diagnostic specificity (100%) and accuracy (97%) were higher using MMSS as compared to conventional cytology in which case specificity was 83.6% and accuracy was 82.1%.

Conclusions: Cytological grading system based on MMSS allowed accurate and reproducible diagnosis compared to the standard histopathological diagnosis. It is essential to differentiate non-proliferative lesions from proliferative lesions as the line of treatment and prognosis varies.

## Introduction

In India, cancer of the breast is the most common malignancy among women. There has been an increase in breast cancer incidence worldwide.

Fine-needle aspiration cytology (FNAC) is a part of the triple assessment of breast lumps. Shahla Masood, in the year 2005, developed a cytological grading system to categorize breast lesions based on features like cellular arrangement, degree of cellular pleomorphism, Anisonucleosis, presence of myoepithelial cells, nucleoli, and type of chromatin pattern [1]. A total score between 6 and 10 was indicative of non-proliferative breast disease (NPBD), 11 and 14 of proliferative breast disease (PBD) without atypia, 15 and 18 of PBD with atypia, and between 19 and 24 of carcinoma in situ and invasive cancer. Application of this scoring system on aspirates in a stepwise manner can help in the selection of cases suitable for biopsy.

A Modified Masood scoring system (MMSS) was proposed in the year 2011 by Nandini et al. to improve the diagnostic accuracy of NPBD and PBD with and without atypia, as the prognosis and treatment of these cases vary. The NPBD in this scoring system was represented by scores between 6 and 8 and PBD without atypia by scores between 9 and 14 [2].

The present study was undertaken to cytologically categorize the breast lesions as per MMSS criteria and conventional method. This was followed by comparing the diagnosis with the histopathological examination (HPE), which is the gold standard.

## Materials and methods

The present study was conducted in the Cytology section of the Department of Pathology, Christian Medical College and Hospital, Ludhiana, India. This was a study done on 67 patients who presented with palpable breast lumps and underwent FNAC followed by an HPE by core-cut biopsy. It was a prospective study done over a period of one and a half years from November 2012 to May 2014.

Method of collection of data

Clinical information like age, presenting features, examination findings of the breast lump, and other investigations were noted from investigation forms (both cytology and histopathology).

Inclusion Criteria

All patients with palpable breast lumps undergoing FNAC and biopsy for HPE were included in the study.

Exclusion Criteria

Inflammatory lesions and breast lesions that were not palpable clinically were not included in the study.

FNAC procedure

Patients were informed about the procedure and consent was taken. As per the standard guidelines under all aseptic conditions the FNAC procedure was carried out. Minimum one air-dried and two wet smears were made. Wet smears were immediately fixed in 95% alcohol for 15 to 30 minutes. Wet-fixed smears stained by hematoxylin and eosin staining methods. Air-dried smears were fixed in methanol and stained with May-Grunwald Giemsa (MGG) stain [3].

Conventional cytological diagnosis was made based on cytomorphological features. This was followed by a diagnosis made according to the MMSS grading system.

Values ranging from 1 to 4 were assigned to each criterion. The details are given in Table 1.

**Table 1:** Modified Masood Scoring System for fine-needle aspiration

Cellular arrangement	Monolayer	Nuclear overlapping	Clustering	Loss of cohesion
Cellular pleomorphism	Absent	Mild	Moderate	Marked
Myo-epithelial cells	Many	Moderate	Few	Absent
Anisonucleosis	Absent	Mild	Moderate	Severe
Nucleoli	Absent	Micro-nucleoli	Micro and/or rare macro-nucleoli	Predominantly macro-nucleoli
Chromatin clumping	Absent	Rare	Occasional	Frequent
Score	1	2	3	4

The cells were assessed for arrangement and size. The nuclei were assessed for size, nucleoli, and chromatin clumping. The presence of myoepithelial cells was seen and a score ranging from 6 to 24 was given and categorized accordingly (Table 2).

**Table 2:** The cytological diagnosis was divided into four categories and was made based upon the sum of scores as per individual values

Score	Diagnosis
6-8	Non-proliferative breast disease
9-14	Proliferative breast disease without atypia
15-18	Proliferative breast disease with atypia
19-24	Cancer
-	Miscellaneous/inconclusive

Conventional cytological evaluation and evaluation of smears based on scoring by MMSS was done personally, the results thus obtained were compared with the histopathological findings. Later, statistical analysis was done using Epi-data 6.1 analysis to examine the degree of correlation between the cytological and histopathological diagnosis.

The concordance, diagnostic accuracy, sensitivity, specificity, the positive and negative predictive values of the MMSS were calculated.

## Results

**Figure 1:**
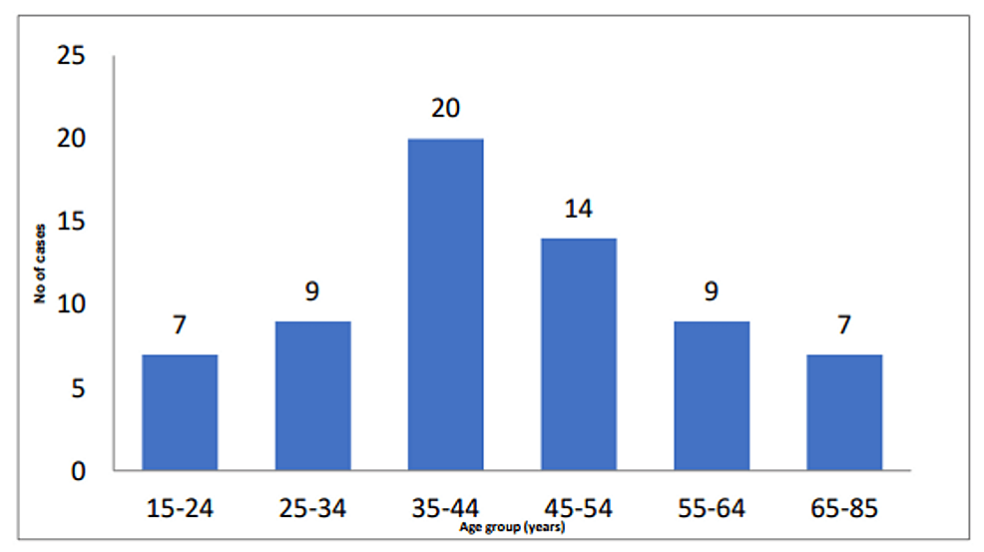
Age-wise distribution of patients included in the study

The majority of the cases were seen in the third to the fourth decade (Figure 1).

**Figure 2:**
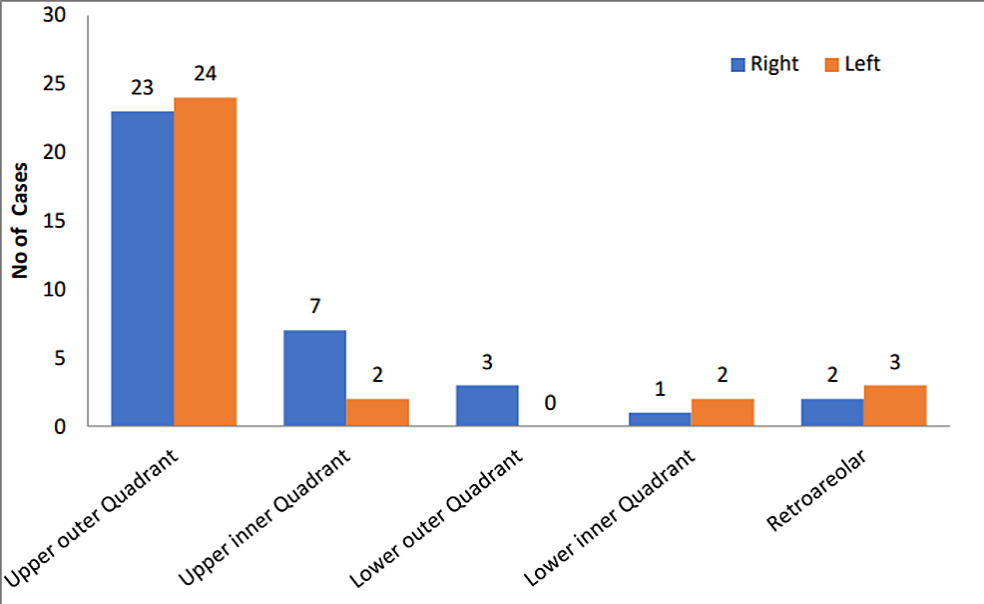
Distribution of patients according to region and side of involvement

The majority of patients were females (66; 98%). The incidence of involvement of the right breast was slightly more as compared to left breast (53.7% versus 46.3%). The upper outer quadrant was the most commonly involved region (70%) (Figure 2).

The majority (62; 92.5%) of the breast lumps examined were firm to hard in consistency. The margins were ill-defined in 35 (52.2%) patients. The lumps were mobile in 32 (47.7%) and non-mobile in 35 (52.2%) patients. Nipple retraction was seen in four (5.9%) patients. Axillary lymphadenopathy was seen in seven (10.4%) patients.

**Figure 3:**
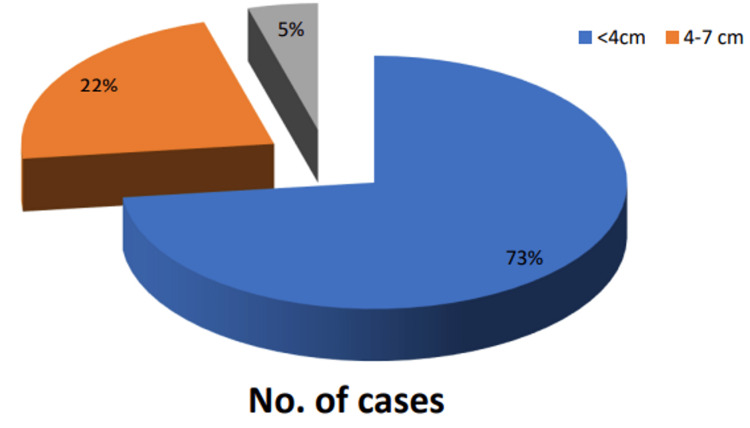
Size variation of breast lesions

The size of breast lesions in most patients (73%) was less than 4 cm in size. The lesion size of more than 7 cm was seen in three cases (Figure 3).

The majority of cases (32;47.7%) in MMSS criteria belonged to Group IV. The most common breast cancer seen was infiltrating ductal carcinoma (30 cases) and there was one case each of mucinous carcinoma and malignant phyllodes tumor. Group II breast lesions comprised 24 (35.8%) cases (fibroadenoma - 18 cases and fibrocystic breast disease - six cases). While Group III category had three (4.7%) cases (fibroadenoma with atypia - two cases and suspicious of carcinoma - one case). There were four (5.9%) cases in Group I (mastitis - two cases and one case each of gynaecomastia and epidermal inclusion cyst). Group V had four (5.9%) cases; all cases had pauci-cellular smears and were reported inconclusive. The detail is shown in Table 3.

**Table 3:** Group-wise distribution by Modified Masood Scoring System

GROUP	No of Cases	Percentage (%)
I (Non proliferative)	04	5.9
II (PBD without atypia)	24	35.8
III (PBD with atypia)	03	4.7
IV (carcinoma)	32	47.7
V (Miscellaneous/inconclusive)	04	5.9
Total	67	100%

Comparison of lesions as per diagnosis is based on conventional cytology, MMSS, and HPE. Evaluation of cytological diagnosis made as per conventional cytology and MMSS were compared with histopathological diagnosis taken as the gold standard. It was observed that there was a higher concordance rate between MMSS and HPE diagnosis in almost all groups (especially in Group II and IV) as compared to diagnosis made on conventional cytology (Table 4).

**Table 4:** Comparison table of lesions diagnosed on conventional cytology and MMSS with histopathological examination (HPE) along with their percentage concordance MMSS - Modified Masood Scoring System

Group	Cytological diagnosis	Conventional Cytology	MMSS	HPE	Percentage concordance between Conventional cytology & HPE	Percentage concordance between MMSS & HPE
I	Non-proliferative breast disease	14	04	06	42.8%	66.6%
II	Proliferative breast disease without atypia	15	24	24	62.5%	100%
III	Proliferative breast disease with atypia	04	03	01	25%	33.3%
IV	Carcinoma breast	30	32	36	83.3%	88.8%
V	Non-neoplastic, miscellaneous lesions, and inadequate smears	05	04	00	-	-
Total		67	67	67	-	-

The overall diagnostic specificity and accuracy were higher by using MMSS as compared to conventional cytology. The details are shown in Table 5.

**Table 5:** Percentage of specificity, sensitivity, positive predictive value, negative predictive value, and accuracy of diagnosis made by conventional cytology and MMSS MMSS - Modified Masood Scoring System

	Conventional cytology	MMSS
Sensitivity	66.6%	66.6%
Specificity	83.6%	100%
Positive predictive value	28.5%	100%
Negative predictive value	96.2%	96.8%
Accuracy	82.1%	97.0%

Cytological diagnosis by conventional cytology and scoring based on MMSS was done. The results thus obtained were compared with the histopathological findings. Kappa test for an inter-rater agreement was used for analysis and showed a moderate agreement of 0.66 (66%) in conventional cytology and good agreement of 0.87 (87.5%) with MMSS (Tables 6, 7).

**Table 6:** Comparison of conventional diagnosis with histopathological diagnosis using kappa analysis Kappa test agreement - 0.66

Conventional cytological diagnosis		Histopathological diagnosis			
	I	II	III	IV	Grand total
Groups	Count	Count	Count	Count	Count
I	4	9		1	14
II	1	14			15
III		1	1	2	4
IV				30	30
V	1			3	4
Grand total	6	24	1	36	67

**Table 7:** Comparison of MMSS with histopathological diagnosis using kappa analysis MMSS - Modified Masood Scoring System Kappa test agreement - 0.875

		Histopathological diagnosis				
	I	II	III	IV	V	Grand total
MMSS	Count	Count	Count	Count	Count	Count
I	4					4
II	1	23				24
III		1	1		1	3
IV				32		32
V	1			3		4
Grand total	6	24	1	35	1	67

## Discussion

The present study was done on 67 patients to analyze the cytomorphological features of breast lesions using conventional cytology. Cytological diagnosis based on MMSS and both were compared with histopathological diagnosis taken as the gold standard. The MMSS was used as a tool for distinguishing non-proliferative breast lesions from proliferative ones. An attempt was made to determine the efficacy, feasibility, and applicability of the cytological grading system using MMSS and its correlation with HPE. The results were divided into five categories: (a) NPBD, (b) PBD without atypia, (c) PBD with atypia, (d) carcinoma, and (e) miscellaneous lesions/inconclusive smears. Accuracy, sensitivity, specificity, and predictive values were calculated.

There are several reports of the performance of Masood’s criteria and MMSS on FNAC with favorable results showing concordance with histopathological diagnosis ranging from 89% to 93.2% [1,4,5]. The percentage of concordance with the cytological grading system based on MMSS in our study was 72.2% (Table 8).

**Table 8:** Various studies and their overall concordance with histopathological diagnosis

Study done by	% of cytological and histopathological diagnosis agreement
Masood et al., 1991 (MSS criteria) [4]	89.0%
Sniege and Staerkel, 1992 (MSS criteria) [5]	93.2%
Nandini et al., 2011 (MMSS criteria) [2]	98.5%
Present study (MMSS criteria)	72.2%

There have been various cytomorphological grading systems of which Masood scoring and MMSS have been considered as one of the sensitive scoring systems in differentiating NPBD, PBD with and without atypia [6,7]. This differentiation into NPBD and PBD with and without atypia is important as various studies have suggested that NPBD has mild risk (1.5-2 times) and PBD with atypia has moderately increased risk (4-5 times) of breast cancer. Patients who have carcinoma in situ have an 8-10 times the risk of developing breast cancer [1,5]. Therefore it is necessary that an accurate diagnosis is offered.

Non-proliferative breast disease (Group I)

The current study showed 14/67 (20.9%) cases diagnosed by conventional cytology as compared to 6/67 cases by HPE. The concordance of conventional cytology with histopathology in this category was 42.8%. Hence, by application of MMSS criteria, a better concordance (66.6% vs 42.8%) was seen with HPE as compared to conventional diagnosis.

The study done by Nandini et al. [2] showed a concordance of 95% cases and the findings observed by Nandini et al. in this category were comparable to the present study

Two studies done by Masood et al., using Masood criteria showed concordance of 85% and 95% with HPE in the non-proliferative category [1,8].

PBD without atypia (Group II)

In the present study, the total number of cases diagnosed as PBD without atypia according to MMSS criteria were 24/67 (35.8%) patients, which were in 100% concordance with diagnosis based on HPE. By conventional cytology, the concordance was 62.5% cases. The application of MMSS showed better concordance (100%) with histopathology in this category.

Nandini et al. documented 97.4% agreement with HPE in this category using MMSS. Other authors like Masood et al. and Mirdha et al. found a high correlation with HPE diagnosis with 88.2% and 97.4% agreement using MSS criteria for diagnosis [2,9].

Sneige and Staerkel, in their study, observed 60% cyto-histopathological correlation in PBD without atypia using Masood’s scoring system and concluded that this grading system is more reliable than cytology alone in the identification of proliferative breast lesions with and without atypia and low-grade carcinoma [5].

Proliferative breast lesions with atypia (Group III)

The cases belonging to group III are important to identify as they may lead to malignancy. By using MMSS criteria, 3/67 (33%) cases were diagnosed in this category compared to 1/67 (1.7%) case diagnosed as fibroadenoma with atypia by HPE. There were two false-positive cases by MMSS (fibroadenoma with atypia - one case, atypical ductal hyperplasia - one case), on histopathology they were grouped under PBD without atypia (fibroadenoma - one case) and carcinoma category (IDC - one case). The concordance of MMSS with HPE was 33.3%.

By conventional, there was a concordance of 25% of conventional cytological diagnosis with HPE in this category. By application of MMSS, a better concordance of 33% was seen.

Other authors also documented cases of PBD with atypia diagnosed on conventional cytology which turned out to be low-grade carcinoma breast on histopathology [1,2,8,9]. Mirdha et al., in their study, concluded that the application of Masood’s scoring system improves the diagnostic yield and gives additional information by eliminating benign cases [9]. Categorization of lesions in this category is challenging as the cytological features of PBD with atypia, PBD without atypia, and low-grade carcinoma of breast overlap [10]. The percent concordance of various studies with HPE compared to the present study is given in Table 4.

Nandini et al. and Masood et al. achieved 100% and 97% cyto-histopathologic correlation, respectively, in this category by application of MMSS and Masood’s criteria for diagnosis. Whereas present study had a 33% correlation with HPE which was much lower in comparison to the study done by Nandini et al. and Masood et al. in this category [2,8].

Carcinoma breast (Group IV)

On application of MMSS criteria, 32/67 (32%) cases were correctly diagnosed as carcinoma as compared to 36/67 (53.7%) on HPE. There was 88.8% concordance with HPE diagnosis.

By conventional cytology, 30 cases were interpreted as carcinoma breast as compared to 36 cases on histology. The discordance in this category was due to insufficient sampling in three cases, misinterpretation as PBD with atypia in two cases, and benign breast disease in one case. Overall concordance of conventional cytology diagnosis, when compared with histopathology in the present study, was 83.3%.

The results in this category were in accordance with Nandini et al., Masood et al., and Mirdha et al. with 100% cytohistological correlation [2,10,11]. Other authors have shown a high degree of concordance with HPE using other grading systems, ranging from 60% to 90% in this category [7,12-15].

Miscellaneous lesions and inconclusive (Group V)

There were 4/67 cases placed in this category as per MMSS criteria. All the inconclusive/ inadequate cases on HPE were diagnosed as carcinoma. The reason for inadequacy could be due to faulty technique, the inexperience of the aspirator or desmoplastic stroma (especially in lobular carcinoma) in malignancy which was also reported in the study done by Scopa et al. [16].

The study done by Masood et al. had 9/100 (9%) cases with insufficient material. Sudarat et al. and Nandini et al. found 4.2% and 3% cases, respectively, with unsatisfactory smears, i.e., fewer than five epithelial cell groups, which needed further repeat aspiration or core/incisional biopsy for analysis. The rate of inadequate aspiration ranges from 0.7% to 25.3% in studies done by various authors [1,17-20].

In our study, the sensitivity of conventional cytological diagnosis and MMSS was found to be 66.6%. The specificity of MMSS and the conventional cytological diagnosis was found to be 100% and 83.6%, respectively. The accuracy of MMSS was higher, i.e., 96.8% as compared to conventional cytology, i.e., 82.1%. The results of the present study were in accordance with studies done by Nandini et al. [2], Mirdha et al. [9], and Masood et al. [10].

## Conclusions

The conclusion of the present study was that the cytological grading system based on MMSS allowed accurate and reproducible diagnosis compared to the standard histopathological category of NPBD, PBD with and without atypia, and carcinoma. It is essential to differentiate non-proliferative lesions from proliferative lesions as the line of treatment and prognosis varies.
